# A diet containing cod backbone proteins attenuated the development of mesangial sclerosis and tubular dysfunction in male obese BTBR *ob/ob* mice

**DOI:** 10.1007/s00394-023-03227-4

**Published:** 2023-08-07

**Authors:** Maria O’Keeffe, Åge Oterhals, Hrafn Weishaupt, Sabine Leh, Arve Ulvik, Per Magne Ueland, Alfred Halstensen, Hans-Peter Marti, Oddrun Anita Gudbrandsen

**Affiliations:** 1 Dietary Protein Research Group, Centre for Nutrition, Department of Clinical Medicine, University of Bergen, Haukeland University Hospital, 5021 Bergen, Norway; 2 Nofima, Bergen, Norway; 3 Department of Pathology, Haukeland University Hospital, Bergen, Norway; 4 Department of Clinical Medicine, University of Bergen, Bergen, Norway; 5 Bevital AS, Bergen, Norway; 6 Department of Clinical Science, University of Bergen, Bergen, Norway

**Keywords:** Fish proteins, Obesity, Glomerulomegaly, Atlantic cod

## Abstract

**Purpose:**

The obese black and tan, brachyuric (BTBR) *ob/ob* mouse spontaneously develops features comparable to human diabetic nephropathy. The primary aim of the present study was to investigate if a diet containing fish proteins would attenuate or delay the development of glomerular hypertrophy (glomerulomegaly), mesangial sclerosis and albuminuria in obese BTBR *ob/ob* mice.

**Methods:**

Obese BTBR.CgLep^ob^/WiscJ male mice were fed diets containing 25% of protein from Atlantic cod backbones and 75% of protein from casein (Cod-BB group), or casein as the sole protein source (control group). Kidneys were analysed morphologically, and markers for renal dysfunction were analysed biochemically in urine and serum.

**Results:**

The Cod-BB diet attenuated the development of mesangial sclerosis (*P* 0.040) without affecting the development of glomerular hypertrophy and albuminuria. The urine concentration of cystatin C (relative to creatinine) was lower in mice fed the Cod-BB diet (*P* 0.0044).

**Conclusion:**

A diet containing cod backbone protein powder attenuated the development of mesangial sclerosis and tubular dysfunction in obese BTBR *ob/ob* mice, but did not prevent the development of glomerular hypertrophy and albuminuria in these mice.

**Supplementary Information:**

The online version contains supplementary material available at 10.1007/s00394-023-03227-4.

## Introduction

Obesity is an independent risk factor for the development of chronic kidney disease and adversely affects the progression of glomerular hypertrophy and focal segmental glomerulosclerosis [1–3]. Paediatric and adult patients with chronic kidney failure are advised to follow a diet with moderate protein restriction to limit the development of uraemia and other metabolic complications [4], but the recommendation does not distinguish between different sources of proteins. Little knowledge exists about how different types of dietary proteins may affect kidney function, especially in individuals with a high risk of developing impaired kidney function, and studies in animal models may be valuable tools to obtain such information. Fish is an excellent source of proteins and essential amino acids, and intake of fish is associated with a reduced risk of developing kidney disease both in the general population [5] and in patients with type 1 diabetes [6].

The kidneys have a major role in amino acid homeostasis through the synthesis, degradation, filtration, reabsorption and urinary excretion of amino acids and peptides and are important regulators of amino acid and protein metabolism [7]. The essential amino acid tryptophan is metabolised mainly through the kynurenine pathway in the liver [8]; however, a considerable quantity of tryptophan enters the colon and is degraded by gut microbes to a variety of indoles [9]. Whereas several of the kynurenine metabolites are neurotoxic, including quinolinic acid and picolinic acid [8], the indoles are, in general, regarded to be favourable with the exception of the uremic toxin indoxyl-3-sulphate which is a metabolite of indole [9]. Indoxyl-3-sulphate is cleared by the proximal tubules and is found in high concentration in circulation in patients with chronic kidney disease and may predict the renal progression [10]. The circulating concentrations of kynurenine pathway metabolites are altered in patients with chronic kidney disease or diabetic kidney disease secondary to type 2 diabetes [11–13], and concentrations are affected by fish intake [14, 15]. As of yet, the effects of fish intake on indoles including indoxyl-3-sulphate have not been investigated in humans or rodents.

We have previously investigated the effects of fish or fish protein intake in obese Zucker fa/fa rats. These rats are leptin-resistant and spontaneously develop metabolic complications of obesity resembling the human metabolic syndrome, including insulin resistance, mild glucose intolerance, hyperinsulinemia, dyslipidemia and high blood pressure, in addition to proteinuria and focal segmental glomerulosclerosis leading to renal failure [16, 17]. When obese Zucker fa/fa rats were fed diets containing lyophilised fish muscle or a protein powder produced from fish residuals, we observed improved postprandial glucose regulation [18, 19], attenuated development of high blood pressure [20–23], and delayed development of kidney dysfunction [20, 24–26].

An elevated blood pressure is associated with an increased risk of chronic kidney diseases [27], and when investigating the effects of fish protein intake on kidney function in the obese Zucker *fa/fa* rat it is difficult to assess whether any observed effect is a direct effect or if it is secondary to attenuation of blood pressure increase. Therefore, in the present study, we wanted to investigate the effect of fish protein intake in the black and tan, brachyuric (BTBR) *ob/ob* mice, which are leptin-deficient and spontaneously develop obesity [28] but are hypotensive when compared to wildtype BTBR [29]. The BTBR *ob/ob* males spontaneously develop hyperglycaemia before 6 weeks of age and are severely type 2 diabetic [28], and mimic human diabetic nephropathy [29]. As a consequence of obesity and diabetes, the BTBR *ob/ob* male mice develop glomerular hypertrophy and mesangial sclerosis (accumulation of mesangial matrix) when they are 8 weeks old, albuminuria at 9 weeks age and interstitial fibrosis after 12 weeks [29]. To the best of our knowledge, the effects of fish protein intake on the development and severity of obesity-related glomerulopathy and diabetic nephropathy have never before been investigated in BTBR *ob/ob* mice. As a lean control, we used BTBR T^+^ Itpr3^tf^/J mice. Since fish intake is associated with a reduced risk of developing kidney disease in humans [5, 6], the main aim of the present study was to investigate if a diet containing fish proteins would attenuate or delay the development of glomerular hypertrophy (glomerulomegaly), mesangial sclerosis and albuminuria in obese BTBR *ob/ob* mice. The secondary aims were to investigate any effects of Cod-BB on the kynurenine pathway metabolites and indoles produced from tryptophan, and to explore any difference between the obese and the lean BTBR strains with respect to these factors. Our hypothesis was that intake of a diet containing Cod-BB would attenuate the spontaneous development of glomerular hypertrophy, mesangial sclerosis, and albuminuria in the BTBR *ob/ob* mice.

## Methods

### Ethical statement

The study protocol was approved by the National Animal Research Authority (Norway) in accordance with the Animal Welfare Act and the Regulation of animal experiments (Approval No. 23928). All applicable international, national and institutional guidelines for the care and use of animals were followed.

### Preparation of cod backbone protein powder

Atlantic cod (Gadus morhua) was captured in the Norwegian Sea outside Lofoten, Norway, in April 2020. Backbone residuals after the filleting operation were frozen and stored at − 23 °C until preparation at Nofima. The backbones were partly thawed overnight at approximately 15 °C and were coarsely ground, added water (4:1 on weight basis), heated to 85 °C under continuous stirring, and kept at this temperature for 10 min. The heat-coagulated raw material was frozen and lyophilized, and the dried product was milled on a Retsch rotomill (aperture 0.75 mm). The obtained cod backbone powder was stored at ambient temperature until analysis and formulation of the mouse diet.

### Animals

Two mouse experiments were conducted. 16 obese BTBR.CgLep^ob^/WiscJ homozygous male mice (JAX stock #004824) and 16 lean BTBR T^+^ Itpr3^tf^/J male mice (JAX stock #002282) were obtained from The Jackson Laboratory (US). The BTBR T^+^ Itpr3^tf^/J mouse (formerly known as the BTBR T^+^ tf/J mouse) was derived from the BTBR (Black and Tan BRachyury) inbred strain, and the BTBR.CgLep^ob^/WiscJ strain was bred from BTBR T^+^ Itpr3^tf^/J, where the *ob* allele from B6.V-Lepob/J was introgressed. Mice were acclimatised for a minimum of 7 days under these conditions, and during the acclimatisation period, two of the obese BTBR mice died without any demonstrable reason. Two obese BTBR mice in the control group were euthanized during the intervention period due to renal failure and very poor health. Thus, a total of 12 obese BTBR mice and 16 lean BTBR mice were included in the biochemical and morphological analyses. The mice of each strain were stratified based on their date of birth and thereafter randomly allocated to the control group or the Cod-BB group by drawing lots. The mice were housed in GM500 Mouse IVC Green Line (Tecniplast, Buguggiate, VA, Italy) (3–4 mice per cage), with a plastic igloo for shelter and GM500925 powder feeder (Tecniplast), under standard conditions at 23–25 °C and a light–dark cycle of 12 h.

### Diets

Modified semi-purified diets were prepared according to the American Institute of Nutrition’s recommendation for growing laboratory rodents (AIN-93G) [30] with the addition of 1.6 g methionine/kg diet as recommended by Reeves [31] and differed only in their protein sources (Table 1). Both diets contained 20 wt% of proteins. The AIN-93G diet was used instead of the AIN-93 M diet for maintenance containing 15 wt% protein, since the mice were in the growth phase at the start of the intervention. Also, *ob/ob* mice have a reduced skeletal muscle growth when compared to their lean littermates due to a faster rate of protein degradation rather than impaired protein synthesis [32], and it is important to secure sufficient protein intake to maintain the growth rate. Casein was the sole protein source in the control diet. Cod backbone protein powder was added to the Cod-BB diet in an amount providing 25 wt% of total protein, while casein constituted the remaining 75 wt% of protein. Sodium chloride was added to the control diet to compensate for the higher sodium content in the cod backbone protein powder compared to casein, resulting in a sodium content of 0.3% in both diets. All ingredients were purchased from Dyets Inc. (Bethlehem, PA, USA) except casein and NaCl (p.a.) which were purchased from Sigma-Aldrich (Munich, Germany), and cod backbone protein powder which was prepared by Nofima (Bergen, Norway). Diets from the same production batches were used for both the obese and the lean BTBR mice. The diets were stored at − 26 °C, and daily portions were thawed in the morning.

**Table 1:** Composition of the experimental diets

Contents (g/100g diet)	Control diet	Cod-BB diet
Casein^a^	22.79	17.09
Cod backbone protein powder^b^	–	8.23
Cornstarch	49.81	47.47
Sucrose	9.00	9.00
Cellulose	5.00	5.00
Soybean oil	7.00	7.00
t-Butylhydroquinone	0.0015	0.0015
Mineral mix (AIN-93-MX)	3.50	3.50
Vitamin Mix (AIN-93-VX)	1.00	1.00
l-Methionine	0.16	0.16
l-Cystine	0.30	0.30
Choline bitartrate^c^	0.25	0.25
Growth and maintenance supplement^d^	1.00	1.00

*Cod-BB* cod backbone protein powder

^a^Contains 87.8% crude protein, 0.80% fat, 10.4% moisture, 2.7% ash

^b^Contains 60.78% crude protein, 1.2% fat, 4.3% moisture, 31.8% ash

^c^Contains 41% choline

^d^Contains vitamin B12 (40 mg/kg) and vitamin K1 (25 mg/kg) mixed with sucrose (995 g/kg) and dextrose (5 g/kg)

### Design

The diets and the experimental setup were first tested in the lean BTBR mice for 50 days. When the same experimental design was applied in the obese BTBR mice experiment, it became evident that the health of these mice was rapidly declining in both dietary groups and we decided to terminate the intervention after 30 days. All mice were fed ad libitum and had free access to drinking water and Aspen gnawing blocks. The mice in both experiments were around 9 weeks old when intervention was initiated. The feed intake was recorded daily in both mouse experiments. In the obese BTBR mice, the water intake was recorded daily to detect any abrupt increase in drinking, as this would indicate the development of diabetes and/or failing kidney function. In the lean BTBR mice, water intake was measured for 48 h during the last week of intervention. The mice were housed individually in metabolic cages for 4 h for collection of urine, without fasting in advance, at 3–4 days before euthanisation. Urine samples were frozen at − 80 °C until analysis. At the end of the experimental period, the mice were fasted for 4–5 h from 8:30 AM, with free access to drinking water, and were euthanized while anaesthetised with isoflurane (Isoba vet, Intervet, Schering-Plough Animal Health, Boxmeer, The Netherlands) mixed with oxygen. The body length was measured with a ruler, while mice were anaesthetised. Blood was drawn from the heart using a syringe, centrifuged, and serum was frozen at − 80 °C. The left kidney was removed and cut in 1–2-mm-thick transversal slices and fixed in 4% buffered formaldehyde for morphological examinations. The epididymal white adipose tissues (WATepi) from both sides were carefully dissected out and weighed.

The personnel handling the mice and conducting the analyses were blinded to the mice’ group allocation. The mice were handled and euthanized in random order.

### Analyses of diets

Contents of amino acids, fatty acids and energy in diets, and contents of amino acids, total fat, moisture and ash in the cod backbone protein powder were measured by Nofima BioLab (Bergen, Norway). Amino acids were measured by HPLC after hydrolysis in 6 N HCl for 22 h at 110 °C and derivatization with 6-aminoquinolyl-*N*-hydroxysuccinimidyl carbamate, with fluorescence detection of the derivatives with excitation/emission at 250/395 nm [33]. Tryptophan was chemically determined by the method of Miller [34]. Fat content was determined gravimetrically after chloroform/methanol extraction [35]. Moisture content was measured gravimetrically after drying in a forced-air oven at 103 ± 1 °C for 4.5 h [36]. Fatty acid composition of diets was analysed by gas chromatography [37] after lipid extraction as described by Bligh and Dyer [35]. Total ash content was determined gravimetrically after incineration at 550 °C [38]. Dietary caloric content was determined by a bomb calorimeter method in accordance with ISO9831:1998 [39]. Sodium in casein and cod backbone protein powder was quantified using inductively coupled plasma optical emission spectrometry in accordance with ISO 11885:2007 [40] by Eurofins (Moss, Norway).

### Light microscopy and morphometry of kidneys

Fixed kidney slices were processed by standard procedures and embedded in paraffin. Three-micrometre thick sections were stained with periodic acid Schiff. Slides were scanned with ScanScope^®^ XT (Aperio) at 40 × resulting in a resolution of 0.25 µm per pixel. Virtual slides were viewed in ImageScope v12.4. All microscopic investigations were performed in a blinded manner.

Glomeruli were automatically detected and segmented from whole slide images via the HistoCloud tool [41], which is available through the Sarder Lab Slide Analyzer (https://athena.ccr.buffalo.edu/histomics). The resulting annotations were extracted as.json files (one per whole slide image), comprising a total of 6317 detections. In some cases, two adjacent glomeruli were annotated as a single object or annotations were incomplete or fragmented. To automatically remove such cases, each annotation of a putative glomerulus was converted to a binary mask and then subjected to a sequence of morphological opening, distance transformation, and thresholding (using the OpenCV library in python), resulting in the identification of 30 annotations possibly including more than one object or incomplete/fragmented annotations that were removed from downstream analyses. Subsequently, the Python library shapely.geometry was utilised to interpret the remaining 6287 annotations as polygons and to calculate their respective areas.

In order to evaluate differences in mesangial glomerular sclerosis between groups, 50 glomeruli per mouse were randomly selected. The resulting 1400 glomeruli (presented in random order) were classified as (1) with or (2) without mesangial sclerosis, respectively. For the 50 images from each mouse biopsy, the percentage of glomeruli with mesangial sclerosis was calculated and used to compare the mouse groups.

### Analyses in urine and serum

Urine albumin concentration was measured using the LSBioTM Mouse ALB/Serum Albumin ELISA Kit (LS-F10450) from LifeSpan BioSciences, Inc. (Seattle, WA, USA). Urine cystatin C was quantified using the Mouse/Rat Cystatin C Quantikine^®^ ELISA (MSCTC0) from R&D Systems, Bio-Techne, MN. All samples were analysed simultaneously in the same plate from each of the two assays, and the plates were read at 450 nm on a SpectraMax Plus384 Microplate Reader (Molecular Devices). The coefficients of variance (CVs) for these assays were 8.1% and 2.6%, respectively. Urine concentrations of creatinine, uric acid, carbamide and glucose, and serum concentrations of creatinine and carbamide were analysed on the Cobas c111 system (Roche Diagnostics GmbH, Mannheim, Germany) using the CREP2 (Creatinine plus ver.2), UA2 (Uric Acid ver.2), UREAL (Urea/BUN) and GLUC2 (Glucose HK) kits from Roche Diagnostics. The between-day CVs for these analyses on the Cobas system were 1.7–5.5%. Tryptophan, kynurenine, kynurenic acid, xanthurenic acid, vitamin B2 (riboflavin and flavin mononucleotide), and vitamin B6 (pyridoxal 5′-phosphate) were analysed in serum by Bevital AS (Bergen, Norway, http://www.bevital.no) using liquid chromatography combined with tandem mass spectrometry, as previously described [42]. Quinaldic acid [43], picolinic acid [44], quinolinic acid [45], indoxyl-3-sulphate, indole-3-propionic acid, indole-3-lactic acid, indole-3-acetic acid, indole-3-aldehyde and indole-3-acetamide with the corresponding isotope labelled internal standards were added to the previously published assay [42]. All samples were analysed in random order. The assay precision for the above methods corresponded to within-day CV of 2.8–9.5% and between-day CV of 4.9–16.9%, as described in detail elsewhere [42–45].

### Outcomes

The primary outcome was to investigate the effects of dietary intake of a diet containing proteins from backbones from Atlantic cod on the development of glomerular changes and albuminuria in young obese BTBR *ob/ob* mice. The secondary aims were to investigate any effects of Cod-BB on the serum concentrations of kynurenine pathway metabolites and indoles produced from tryptophan and investigate any differences between the obese and the lean BTBR mice.

### Sample size

The present study is the first study to investigate the effects of a diet containing a fish protein powder on the development of kidney dysfunction in BTBR.CgLep^ob^/WiscJ mice. Therefore, data on effect size were not available for sample size calculation or minimally detectable effect sizes for the present study. The study was designed with eight mice per experimental group, based on our previous experience with studies using diets containing proteins from fish on kidney function and markers of kidney dysfunction in obese Zucker fa/fa rats showing significant effects with group sizes of six rats [20, 21, 24–26].

### Statistical analyses

Statistical comparisons of median glomerular sizes or percentages of glomeruli with mesangial sclerosis between groups of mice, employing unpaired, two-tailed Welch’s *t* tests, were conducted by *t* test function in R. All other statistical analyses were conducted using SPSS Statistics version 28 (SPSS, Inc., IBM Company, Armonk, NY, USA). Fasting serum concentrations and urine concentrations (relative to creatinine) were evaluated for normality using the Shapiro–Wilk test, Q–Q plots and histograms. Most variables were not normally distributed; consequently, all variables were log-transformed before parametric statistical tests were performed. The paired samples *T* test was used to detect baseline to endpoint changes within groups, and the independent samples *T* test was used to compare endpoint measurements between dietary groups within strain, and between obese and lean mice fed the control diet. The cut off value for statistical significance was set at a probability of 0.05. Statistical analyses for the energy intake and the water intake between the dietary groups for the individual mouse experiments were not conducted, since they consisted of data from only two cages each.

## Results

### Description of diets, and energy and water intake

The dietary contents of indispensable amino acids were similar between the diets, i.e. deviations were ≤ 0.1 g/100 g diet (Table 2). Eicosapentaenoic acid (EPA) and docosahexaenoic acid (DHA) were found only in the cod-BB diet (Table 2). The energy content was similar in the two diets (difference < 2%, Table 2).

**Table 2:** Dietary contents of indispensable amino acids, n-3 long-chain polyunsaturated fatty acids, and calories

	Control diet	Cod-BB diet
Amino acids (g/100 g diet)		
Histidine	0.55	0.54
Isoleucine	1.0	1.0
Leucine	1.9	1.9
Lysine	1.7	1.6
Methionine	0.73	0.71
Phenylalanine	1.1	1.1
Threonine	0.84	0.79
Tryptophan	0.23	0.20
Valine	1.4	1.3
Fatty acids (g/100 g diet)		
20:5 n-3	ND	0.2
22:5 n-3	ND	ND
22:6 n-3	ND	0.4
Energy (kJ/g diet)	17.72	17.38

Means of two measurements; deviations were < 5% between parallels

*Cod-BB* backbone from cod, *ND* not detected

**Fig. 1:**
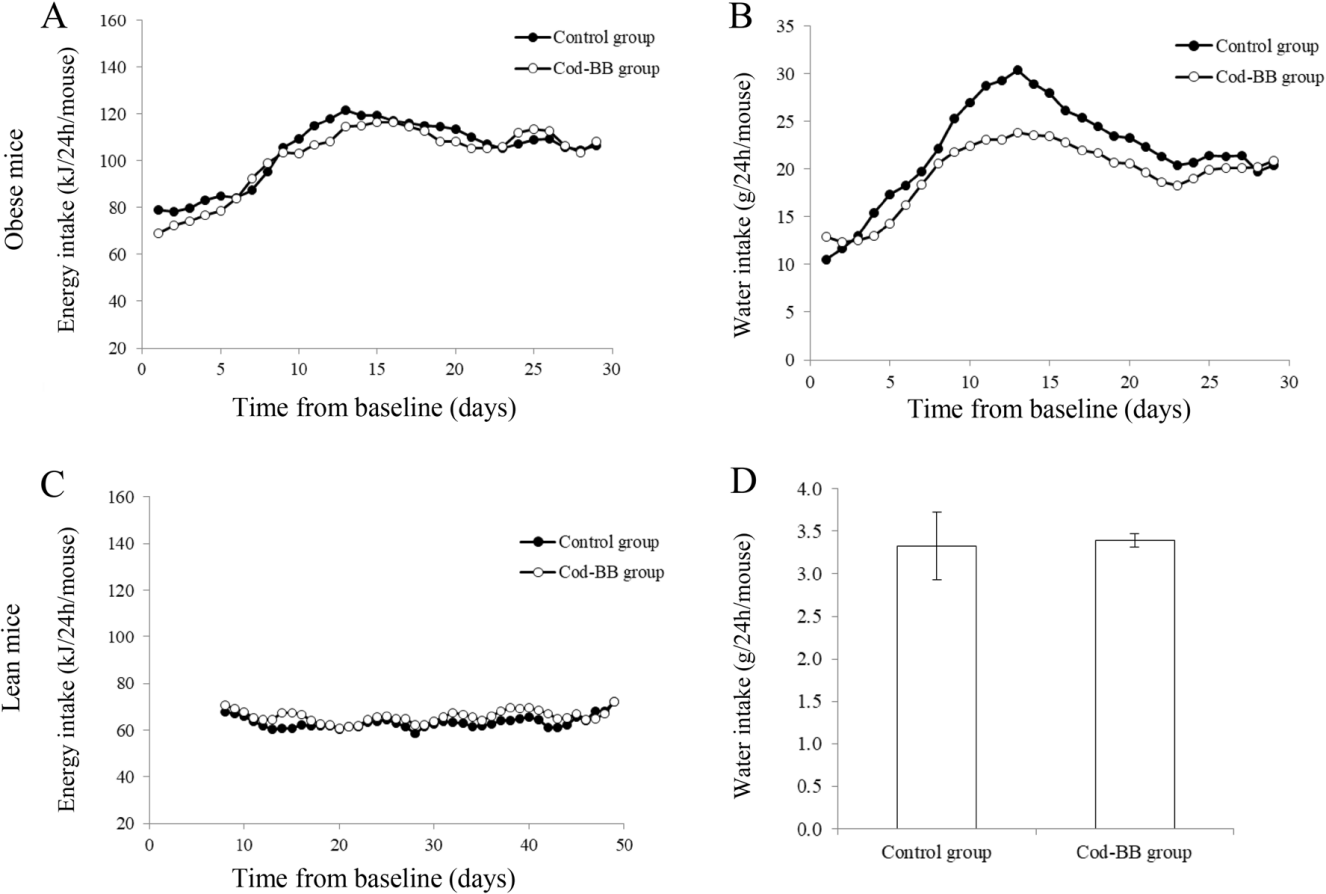
Energy intake (**a**) and water intake (**b**) in obese BTBR mice, and energy intake (**c**) and water intake (**d**) in lean BTBR mice. Energy intake (A, C) and water intake (**b**) are presented in figures as 3-day moving average (to filter out random day to day variations) since registrations were not conducted at the exact same time every day. Water intake in **d** is the average with standard deviation of 48 h recording conducted at the end of the intervention period. Statistical analyses for the energy intake and the water intake between the dietary groups for the individual mouse experiments were not conducted, since they consisted of only two cages from each experimental group

The feed intake was registered daily all through the intervention periods for the obese BTBR mice (Fig. 1a), showing an accumulated average intake of 3147 kJ and 3056 kJ per mouse in the control group and in the Cod-BB group, respectively. The feed intake shows a tendency to increase sharply from 70–80 kJ/24 h to around 115–120 kJ/24 per mouse after 10–11 days and than stabilised at an intake between 105 and 120 kJ/24 h. For the lean BTBR mice, feed intake was registered daily from the 8th day of the intervention until the day of euthanisation (day 50, Fig. 1c), with little variation during the intervention period and showing an accumulated average intake of 2636 kJ and 2751 kJ per mouse in the control group and in the Cod-BB group, respectively.

The water intake was recorded daily for the entire intervention period for the obese BTBR mice (Fig. 1b), showing a trend for increased water intake that peaks after about 10 days of intervention in both dietary groups, which is quite similar to what is seen for the feed intake. Although we have too few observations for conducting statistical testing, the water intake seems to be more pronounced for the mice in the control group compared to the Cod-BB group on the 8th to the 15th day of the intervention. The accumulated water intake for each mouse is also numerically higher in the control group; 670 g as compared to 581 g in the Cod-BB group. The average daily water intake in the lean BTBR mice experiment showed little difference between the dietary groups (Fig. 1d).

### Body weight gain and epididymal adipose tissue weight

**Fig. 2:**
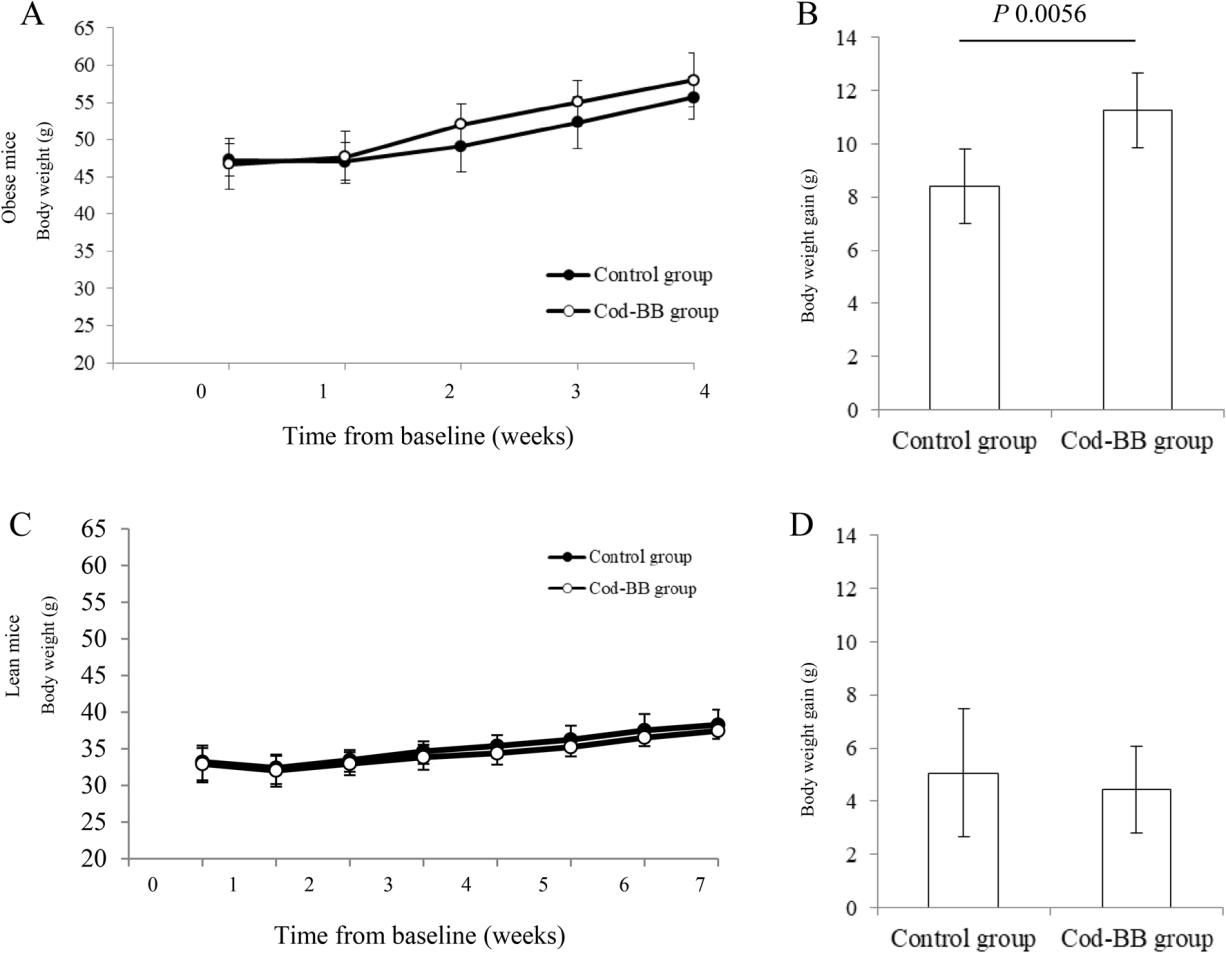
Body weight measured at baseline (week 0), and weekly measurements for obese mice (**a**) and lean mice (**c**) fed the control diet or the Cod-BB diet, and the total body weight gain for obese (**b**) and lean (**d**) mice. Values are geometric means for n 6 in obese experimental groups and n 8 in lean experimental groups, with standard deviations represented by vertical bars, evaluated by independent-samples t test assuming equal variances

The body weight in the obese BTBR mice was similar between the dietary groups at baseline, and after 2, 3 and 4 weeks of intervention (Fig. 2a). The total body weight gain from baseline to endpoint was higher in the Cod-BB group (*P* 0.0056, Fig. 2b), whereas no difference was seen between the groups for the body weight-to-square body length ratio, or for the relative weight of epididymal white adipose tissue (Table 3).

**Table 3:** Body weight-to-square body length ratio and relative weight of epididymal white adipose tissue at endpoint* (geometric means and standard deviations)

	Obese BTBR mice				Lean BTBR mice				*P* diet obese	*P* diet lean	(Control diet)
	Control group		Cod-BB group		Control group		Cod-BB group				
	GM	SD	GM	SD	GM	SD	GM	SD			
Body weight-to-square body length (mg/cm^2^)	508	32	540	34	316	17	309	4	0.13	0.31	1.6 × 10^–8^
Relative WATepi weight (mg/g body weight)	39.4	2.8	39.9	3.4	20.6	2.5	19.5	1.9	0.78	0.37	6.5 × 10^–8^

*P* < 0.05 was considered significant

*Cod-BB* cod backbone protein powder, *GM* geometric mean, *SD* standard deviation, *WATepi* epididymal white adipose tissue.

*Values are shown for *n* 6 mice in the control group and *n* 6 mice in the Cod-BB group in the obese BTBR mouse experiment, and for *n* 8 mice in the control group and *n* 8 mice in the Cod-BB group in the lean BTBR mouse experiment. *P* < 0·05 was considered significant. Groups are compared within each experiment using the independent-samples *t* test assuming equal variances. Control groups in each mouse experiment are compared using the independent-samples *t* test assuming equal variances.

In the lean BTBR mice, no differences were seen for the registered body weight between the Cod-BB group and the control group at baseline or any of the weekly measurements (Fig. 2c), and the total body weight gain (Fig. 2d) and the body weight-to-square body length ratio (Table 3) were similar between the groups. Also, the relative weight of epididymal white adipose tissue was similar between the dietary groups in the lean BTBR mice experiment (Table 3).

When obese and lean BTBR mice fed the control diet were compared, the body weight-to-square body length ratio and the relative epididymal white adipose tissue weight were significantly higher in the obese BTBR mice (Table 3). As expected, the daily intakes of feed and water were numerically higher in the obese BTBR mice, which are hyperphagic due to being leptin-deficient, and had a higher body weight and larger adiposity compared to the lean BTBR mice.

### Morphological examinations of kidneys

**Fig. 3:**
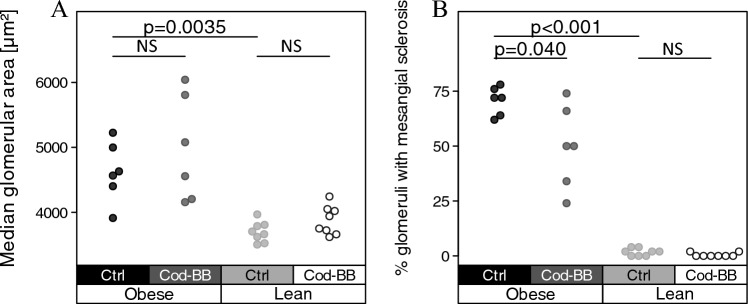
Glomerular sizes and mesangial sclerosis in obese and lean BTBR mice. The strip chart in **a** displays the distribution of median glomerular sizes within each condition; each dot indicates the median size computed for one whole slide image. The strip chart in **b** displays the percentages of glomeruli with mesangial sclerosis across the conditions; each dot represents the percentage for a single mouse kidney based on 50 examined glomeruli. *P* values represent the results of unpaired, two-tailed Welch’s *t* tests. *NS* not significant

The Cod-BB diet did not influence the development of glomerular hypertrophy in the obese BTBR mice (Fig. 3a and Supplemental Fig. 1); however, obese BTBR mice fed the Cod-BB diet had a significantly lower percentage of glomeruli with mesangial sclerosis when compared to the obese mice fed the control diet (Fig. 3b).

Neither the obese nor the lean BTBR mice showed signs of tubular atrophy, interstitial fibrosis, segmental or global glomerulosclerosis. When obese and lean BTBR mice fed the control diet were compared, the median glomerular size was significantly larger (Fig. 3a) and the percentage of glomeruli with mesangial sclerosis was significantly higher (Fig. 3b) in the obese BTBR mice when compared to the lean BTBR mice. Supplemental Fig. 2 shows representative images of normal glomerulus from lean BTBR mice and of glomerulus with mesangial sclerosis from obese/diabetic BTBR mice.

### Markers of kidney function

In the obese BTBR mice, the urine concentration of cystatin C (relative to creatinine) was significantly lower in the Cod-BB group compared to the control group (Table 4), thus indicating an attenuation of tubular dysfunction. The urine concentrations (relative to creatinine) of the nitrogen-containing compounds albumin, carbamide and uric acid were similar between the Cod-BB group and the control group (Table 4). The urine glucose concentration showed a large variation between the obese BTBR mice in both dietary groups (4.5–1395 mmol/mmol creatinine in the control group, 3.0–585 mmol/mmol creatinine in the Cod-BB group), probably due to the mice not developing diabetes at the same time and to a similar degree, and did not reach statistical significant difference when groups were compared (Table 4). The serum concentrations of creatinine and carbamide were similar between the dietary groups in the obese experiment (Table 4).

**Table 4:** Markers of kidney dysfunction measured in urine or serum* (geometric means and standard deviations)

	Obese BTBR mice				Lean BTBR mice				*P* diet obese	*P* diet lean	*P* strain
	Control group		Cod-BB group		Control group		Cod-BB group				(control diet)
	GM	SD	GM	SD	GM	SD	GM	SD			
Urine											
Albumin (mg/mmol creatinine)	219	98	235	68	29.9	8.5	11.8	2.6	0.77	8.2 × 10^–5^	8.4 × 10^–7^
Cystatin C (µg/mmol creatinine)	192	94	117	21	53	16	49	19	0.0044	0.64	5.3 × 10^–5^
Carbamide (mmol/mmol creatinine)	653	36	650	37	384	38	344	54	0.90	0.050	4.6 × 10^–8^
Uric acid (µmol/mmol creatinine)	703	91	659	117	418	85	502	162	0.46	0.18	3.9 × 10^–4^
Glucose (mmol/mmol creatinine)	159	588	44	243	0.38	0.08	0.39	0.07	0.36	0.87	5.3 × 10^–6^
Serum											
Creatinine (µmol/l)	8.7	1.5	10.5	1.6	7.5	2.2	6.1	1.5	0.090	0.20	0.33
Carbamide (mmol/l)	8.5	1.4	9.0	2.3	6.6	1.7	5.4	1.2	0.52	0.091	0.056

*P* < 0.05 was considered significant

*Cod-BB* cod backbone protein powder, *GM* geometric mean, *SD* standard deviation

*Values are shown for *n* 6 mice in the control group and *n* 6 mice in the Cod-BB group in the obese BTBR mouse experiment, and for *n* 8 mice in the control group and *n* 8 mice in the Cod-BB group in the lean BTBR mouse experiment. *P* < 0·05 was considered significant. Groups are compared within each experiment using the independent-samples t test assuming equal variances. Control groups in each mouse experiment are compared using the independent-samples *t* test assuming equal variances

The urine albumin concentration (relative to creatinine) was significantly lower (*P* 8.2 × 10^–5^) and the relative urine carbamide concentration tended to be lower (*P* 0.050) in lean BTBR mice fed the Cod-BB diet when compared to the control group (Table 4). The relative urine concentrations of cystatin C, uric acid and glucose, as well as the serum concentrations of creatinine and carbamide, were similar between the lean BTBR mice groups (Table 4).

The urine concentrations (relative to creatinine) of albumin, cystatin C, carbamide, uric acid and glucose were significantly higher in the obese BTBR mice compared to the lean BTBR mice fed the control diet (Table 4). The serum creatinine and carbamide concentrations were similar between the obese and the lean BTBR mice.

### Tryptophan kynurenine pathway metabolites and cofactors, and indoles

Obese mice fed Cod-BB diet had a higher tryptophan concentration when compared to their controls, with no differences between the obese groups for kynurenines or indoles (Table 5). In lean mice fed Cod-BB diet, the kynurenine/tryptophan ratio was higher, and the quinaldic acid serum concentration was lower when compared to the control group, with similar concentrations of kynurenines and indoles between the groups (Table 5). When obese and lean control mice were compared, the obese mice had higher serum concentrations of tryptophan, picolinic acid, quinolinic acid, riboflavin, flavin mononucleotide, pyridoxal 5′-phosphate and of all the measured indoles, and lower xanthurenic acid concentration, with no differences between the groups for the other kynurenines (Table 5).

**Table 5:** Tryptophan, kynurenine pathway metabolites and cofactors, and indoles measured in serum* (geometric means and standard deviations)

	Obese BTBR mice				Lean BTBR mice				*P* diet obese	*P* diet lean	*P* strain
	Control group		Cod-BB group		Control group		Cod-BB group				(control diet)
	GM	SD	GM	SD	GM	SD	GM	SD			
Tryptophan (µmol/l)	120.1	9.9	139.1	16.3	81.7	9.4	80.6	15.1	0.031	0.86	1.7 × 10^–5^
Kynurenine (µmol/l)	0.68	0.12	0.89	0.28	0.51	0.18	0.64	0.13	0.076	0.098	0.056
Kynurenine/tryptophan ratio^a^	5.7	0.9	6.4	2.7	6.3	1.6	7.9	1.2	0.47	0.038	0.37
Kynurenic acid (nmol/l)	92.5	10.2	112.0	28.3	88.3	26.9	59.4	39.9	0.12	0.075	0.72
Quinaldic acid (µmol/l)	1.3	0.8	1.4	1.2	3.1	3.8	1.3	0.6	0.83	0.019	0.050
Xanthurenic acid (nmol/l)	28.2	6.7	36.4	14.6	78.1	50.8	72.7	134.4	0.18	0.83	7.7 × 10^–4^
Picolinic acid (nmol/l)	271	78.7	302	74.6	157	27.1	134	31.1	0.53	0.14	1.6 × 10^–3^
Quinolinic acid (nmol/l)	233	49	323	120	157	34	102	65	0.10	0.050	0.0074
Riboflavin (nmol/l)	146.5	15.5	143.4	30.1	83.5	57.1	68.5	15.8	0.85	0.25	0.015
Flavin mononucleotide (nmol/l)	74.4	38.5	84.2	33.5	33.6	8.3	34.1	14.0	0.60	0.93	9.3 × 10^–4^
Pyridoxal 5′-phosphate (nmol/l)	769	69	808	107	465	91	483	89	0.44	0.69	8.0 × 10^–5^
Microbiota-derived indoles											
Indoxyl-3-sulphate (µmol/l)	7.4	5.7	7.4	3.9	2.0	0.5	1.5	1.4	0.98	0.23	8.2 × 10^–5^
Indole-3-propionic acid (µmol/l)	0.60	0.58	0.49	0.64	< LOD		< LOD		0.70	N/A	N/A
Indole-3-lactic acid (µmol/l)	1.4	0.6	1.4	0.3	0.5	0.1	0.5	0.1	0.81	0.63	3.6 × 10^–5^
Indole-3-acetic acid (µmol/l)	2.6	0.8	2.6	0.5	0.5	0.1	0.5	0.1	0.97	0.47	3.3 × 10^–8^
Indole-3-aldehyde (nmol/l)	87.8	30.0	86.6	41.8	30.7	13.2	32.3	7.5	0.95	0.76	7.9 × 10^–5^
Indole-3-acetamide (nmol/l)	1.2	0.1	1.3	0.2	< LOD		< LOD		0.69	N/A	N/A

*P* < 0·05 was considered significant. Groups are compared within each experiment using the independent-samples *t* test assuming equal variances. Control groups in each mouse experiment are compared using the independent-samples *t* test assuming equal variances

*Cod-BB* cod backbone protein powder, *GM* geometric mean, *N/A* not available, *LOD* level of detection, *SD* standard deviation

*Values are shown for *n* 6 mice in the control group and *n* 6 mice in the Cod-BB group in the obese BTBR mouse experiment, and for *n* 8 mice in the control group and *n* 8 mice in the Cod-BB group in the lean BTBR mouse experiment.

^a^Kynurenine × 1000/tryptophan

## Discussion

In the present article, we show that consumption of a diet containing protein powder prepared from cod backbones resulted in a lower percentage of glomeruli with mesangial sclerosis, which is a hallmark of diabetic nephropathy, in obese and diabetic BTBR *ob/ob* mice. In addition, the development of tubular dysfunction was attenuated in these mice, as indicated by the lower urine cystatin C concentration. Cod-BB diet did not prevent or delay the development of glomerular hypertrophy and albuminuria in obese BTBR *ob/ob* mice, which spontaneously develop diabetic nephropathy [29]. We also show that serum concentrations of kynurenine pathway metabolites and indoles produced from tryptophan were not affected by the Cod-BB diet in obese or lean BTBR mice. As expected, the differences between obese and lean BTBR mice were substantial with regard to most measured parameters; the obese mice had more epididymal white adipose tissue, larger glomerular sizes, mesangial sclerosis, more evolved albuminuria, and higher urinary concentrations (relative to creatinine) of cystatin C, carbamide, uric acid and glucose than the lean mice in this study.

Mesangial sclerosis develops in diabetic nephropathy, and glomerular hypertrophy is a common histological change in the kidneys related to obesity and diabetes [46]. Here, we wanted to investigate the potential protective effect of a fish protein powder on the development of glomerular hypertrophy, mesangial sclerosis and albuminuria in young obese BTBR mice with diabetic nephropathy. The effects of fish protein intake on glomerular abnormalities and albuminuria have never before been investigated in obese BTBR *ob/ob* mice, and in the present study, we chose to test a cod protein powder since cod is a commercially available and commonly consumed fish. Previous studies from our research group have shown that diets containing cod proteins improved postprandial glucose regulation [19], delayed the development of kidney dysfunction [24] and lowered concentrations of markers of kidney dysfunction and reduced urinary loss of amino acids [26] in obese Zucker fa/fa rats. Moreover, strong findings from other research groups show that dietary cod proteins also improve glucose tolerance and insulin sensitivity in Wistar rats [47–49]. Thus, we presumed that cod proteins could have the potential to attenuate or delay the progress of kidney dysfunction when fed to obese BTBR mice, which develop glomerular hypertrophy and mesangial sclerosis at 8 weeks and albuminuria at 9 weeks [29]. In the present study, the obese BTBR mice were around 9 weeks old at the start of the intervention, and the morphological examinations revealed that the Cod-BB diet did in fact attenuate, or possibly reversed, the development of mesangial sclerosis, but did not affect the development of glomerular hypertrophy, albuminuria or adiposity after 30 days of intervention. In addition, the significantly lower urine cystatin C concentration in the obese Cod-BB group indicates attenuation of the development of renal dysfunction, since urine cystatin C is a specific marker of tubular dysfunction [50] and is associated with renal dysfunction in patients with obesity [51] and in diabetic Zucker fa/fa rats [52].

Contrarily to what was observed in the obese BTBR mice, the Cod-BB diet resulted in lower urine albumin concentration (relative to creatinine) in the lean BTBR mice. Since little knowledge exists about the renal function in the lean BTBR T^+^ Itpr3^tf^/J mice, this finding may be of substantial importance as the median relative albumin concentration in the lean control group (29.9 mg/mmol creatinine) is within the definition of microalbuminuria (30–299 mg albumin/g creatinine [53], corresponding to 3.4–34 mg albumin/mmol creatinine). Although other markers of kidney function, including urine cystatin C, carbamide and glucose, were similar between the experimental lean dietary groups, the Cod-BB diet shows promise as it may prevent or delay the development of albuminuria in this mouse strain.

The Cod-BB diet had only minor effects on serum concentrations of kynurenines, both in the obese and in the lean BTBR mice. This is in contrast with findings in diabetic patients with coronary artery disease where lean fish intake affected kynurenine metabolite concentrations [14], but is in line with our previous studies showing that cod muscle intake did not affect circulating concentrations of kynurenines in obese Zucker fa/fa rats [24] or in non-diabetic adults with overweight or obesity [15]. The effects of fish intake on circulating concentrations of indoles have not formerly been explored; however, since lean fish intake modulated gut microbiota in a recent clinical trial [54], and since fish is a valuable dietary source for tryptophan, intake of fish could be expected to affect gut microbe indole production and hence the concentrations of indoles in circulation. Contrarily to expectations, consumption of the Cod-BB diet did not affect serum concentrations of indoles in any of the mouse strains investigated. A lower serum concentration of indoxyl-3-sulphate could be anticipated in the obese BTBR mice fed the Cod-BB diet since development of mesangial sclerosis and tubular dysfunction was attenuated when compared to corresponding control group, however, no difference was seen between the dietary groups. Taken together, Cod-BB intake had only a marginal effect on the metabolism of tryptophan through the kynurenine pathway and the degradation of tryptophan by gut microbes to indoles.

When comparing the obese and the lean BTBR mice fed the control diet, the serum concentrations of tryptophan, of the kynurenines picolinic acid and quinolinic acid, of the cofactors involved in the kynurenine pathway, i.e. vitamins B2 and B6, and of the six measured indoles (indoxyl-3-sulphate, indole-3-propionic acid, indole-3-lactic acid, indole-3-acetic acid, indole-3-aldehyde and indole-3-acetamide) were higher, whereas the xanthurenic acid concentration was lower, in the obese mice when compared to the lean mice. We propose three possible explanations for these between-strain differences. First, the differences between the strains may be a consequence of the higher feed intake, and thus a higher intake of proteins, in the obese BTBR mice, which are leptin-deficient and hence hyperphagic, and thereby ingesting higher amounts of both the precursor tryptophan and the vitamin cofactors involved in the kynurenine and the indole pathways [9, 55, 56]. Secondly, the higher concentrations of the kynurenines picolinic acid and quinolinic acid, which are neurotoxic [8], in the obese BTBR mice could indicate a higher metabolism of tryptophan down-stream of kynurenine since the kynurenine–tryptophan ratio was similar between the groups. This is in line with findings showing that the kynurenine pathway of tryptophan degradation is upregulated in human obesity [57]. Thirdly, higher circulating concentrations of kynurenine pathway metabolites have been demonstrated in patients [13, 58–60] and rats [59] with impaired renal function. The higher quinolinic acid concentration was suggested to be a consequence of lower aminocarboxymuconate-semialdehyde decarboxylase activity in the liver, thus directing the kynurenine pathway metabolites towards the NAD pathway rather than towards the glutarate pathway [59]. Our findings of higher concentrations of picolinic acid and quinolinic, and lower xanthurenic acid, in the obese BTBR mice compared to the lean BTBR mice, support this proposal.

The lean BTBR mice (BTBR T^+^ Itpr3^tf^/J mice) have a non-synonymous polymorphism in the *Kmo* gene which encodes kynurenine 3-hydroxylase [61]. This enzyme catalyses the conversion of kynurenine to 3-hydroxykynurenine, and the higher concentration of kynurenic acid in the prefrontal cortex in these mice compared to C57Bl/6J mice [62] suggests that tryptophan metabolism is directed towards the production of the glutamate antagonist kynurenic acid, and possibly towards anthranilic acid although this was not measured, rather than towards 3-hydroxykynurenine. A search for single nucleotide polymorphisms in the *Kmo* gene has not, to the best of our knowledge, been conducted in the BTBR *ob/ob* mice; however, the similar serum kynurenic acid concentration in the obese and lean BTBR mice in the present study may indicate that the activity of kynurenine 3-hydroxylase is similar between these strains.

The Cod-BB diet did not affect the development of adiposity of the obese mice, thus the larger body weight gain combined with a similar body weight-to-square body length ratio and similar energy intake between Cod-BB and control obese groups indicate a better utilisation of proteins for muscle building in the Cod-BB group. This is comparable to previous observations where obese Zucker *fa/fa* rats fed cod proteins had lower loss of nitrogen-containing compounds, including amino acids, and higher body weight gain but similar body weight-to-square body length ratio and energy intake compared to a casein-fed control group [26].

Dietary intervention studies in mouse models that strongly resembles many of the characteristics of human diabetic kidney disease, such as the obese BTBR mouse [29], are highly relevant. By testing a diet where cod protein powder replaced only 25% of the control protein casein, instead of total replacement of casein as is used in many dietary protein intervention rodent studies, this diet design is more relevant for human nutrition. Fish residual proteins contain motifs with angiotensin-I converting enzyme (ACE) inhibitor properties [25, 63], which is of interest as treatment with ACE inhibitor drugs are used to delay the progression of chronic kidney disease and amend proteinuria in patients with kidney disease [64]. Thus, these bioactive peptides may play an important role in the observed effects of the Cod-BB diet on the kidneys in the present study. The fish protein powder that was tested in the present study was produced from cod backbones obtained after the filleting operation. The side-streams head, backbone, skin, and cuttings are valuable food grade raw materials with a high protein content and potential for upcycling into food applications [65]. The backbone fraction contains substantial amount of muscle protein, and due to a low fat content, it may be directly heat-treated, dried, and milled to a high protein fish powder. The simple processing route makes fish backbones an attractive material for food product development and testing of bioactive compounds. Development of novel food applications is also highly relevant from a sustainability perspective since large amounts of protein-rich fish residuals are produced by the world’s fish filleting industry, but only a small quantity is used for human consumption [66].

A limitation of the study is the relatively short intervention period in the obese BTBR mouse experiment. Since the health of the obese mice in both dietary groups was deteriorating, we decided to terminate the intervention after 30 days. A longer intervention period would probably result in more pronounced kidney damage, and possibly larger differences between dietary groups.

To conclude, a diet containing cod backbone protein powder attenuated the development of mesangial sclerosis and tubular dysfunction in young BTBR *ob/ob* mice, without preventing the development of obesity-related glomerular hypertrophy and albuminuria in obese mice, but prevented microalbuminuria in lean BTBR mice. Further studies should be conducted to identify the active components in cod backbone proteins and elucidate mechanisms behind the observed effects in the present study, and protein fractions from other fish species and fractions of fish including fillet should be investigated both in animal models and as dietary supplements in clinical studies.

### Supplementary Information

Below is the link to the electronic supplementary material.

Supplementary file1 (PDF 21 KB)

Supplementary file2 (JPG 805 KB)

Supplementary file3 (PDF 1123 KB)
