# Supplementary figures and images for: A diet containing cod backbone proteins attenuated the development of mesangial sclerosis and tubular dysfunction in male obese BTBR ob/ob mice
Source: Eur J Nutr. 2023 Aug 7;62(8):3227–40. doi: 10.1007/s00394-023-03227-4 (PMC10611847; doi:10.1007/s00394-023-03227-4)

Glomerular area [ $\mu\text{m}^2$ ]

15000  
10000  
5000

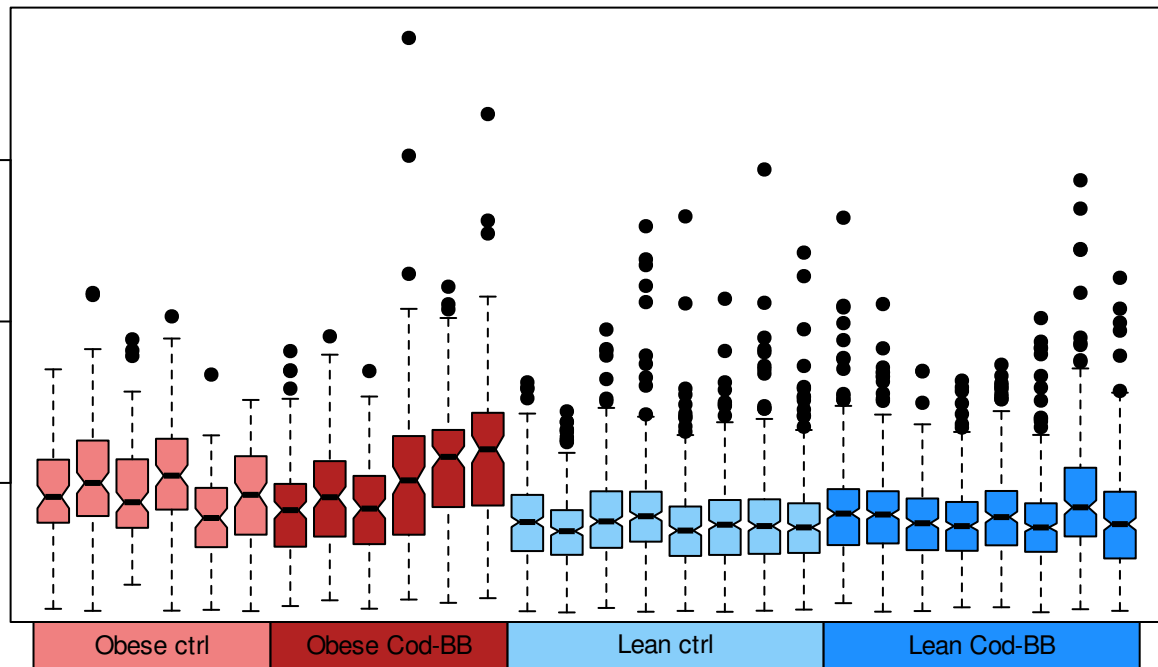

Supplement: Supplementary file 1 — Supplementary file1 (PDF 21 KB) [file 394_2023_3227_MOESM1_ESM.pdf]

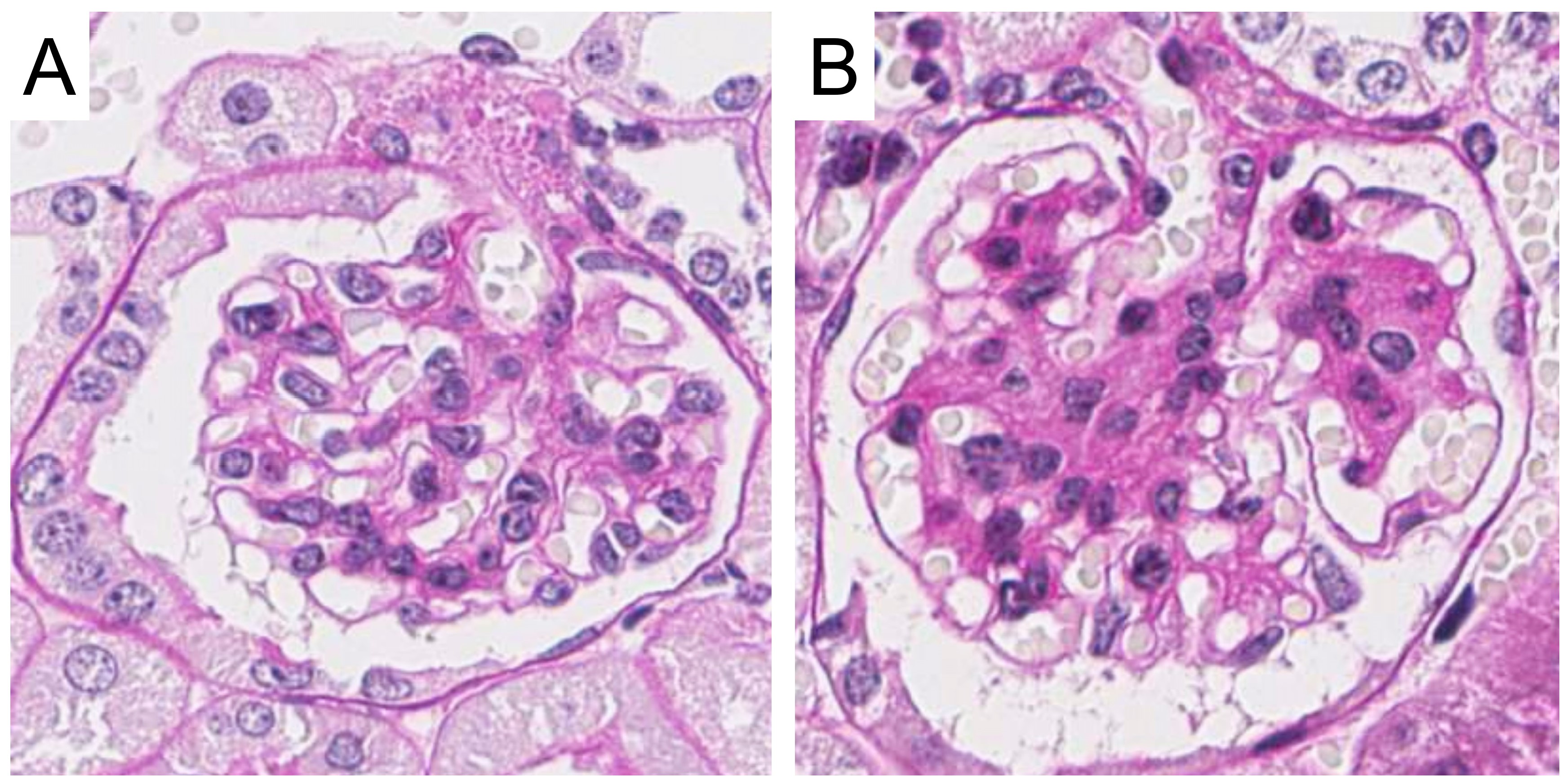

Supplement: Supplementary file 2 — Supplementary file2 (JPG 805 KB) [file 394_2023_3227_MOESM2_ESM.jpg]
